# Bone Mineral Density and Bone Quality Trends in a Child on Steroid Therapy Who Developed a Vertebral Fracture: A Case Report

**DOI:** 10.7759/cureus.82675

**Published:** 2025-04-21

**Authors:** Jun Aoyagi, Takahiro Kanai, Takane Ito, Marika Ishii, Toshihiro Tajima

**Affiliations:** 1 Department of Pediatrics, Jichi Medical University, Shimotsuke, JPN

**Keywords:** alkaline phosphatase, antiresorptive treatment, bone quality, bone strength, bone turnover biomarkers, child, glucocorticoid-induced osteoporosis, tartrate-resistant acid phosphatase 5b, undercarboxylated osteocalcin, vertebral fractures

## Abstract

Glucocorticoids (GCs) are commonly used to treat kidney problems in children and usually work well, but they can sometimes cause bone thinning, which may lead to fractures in the spine. Despite this, there is currently no established clinical approach for managing GC-induced osteoporosis (GIOP) in pediatric patients, highlighting the need for more data. Bone strength reflects both bone mineral density (BMD) and bone quality, with BMD assessed by X-ray and bone quality evaluated through serum or urine bone turnover markers (BTMs). In this case, a seven-year-old girl diagnosed with Henoch-Schönlein purpura nephritis was monitored over a two-year period during steroid treatment. Her BMD and serum BTMs, including alkaline phosphatase (ALP), tartrate-resistant acid phosphatase 5b (TRACP-5b), and undercarboxylated osteocalcin (ucOC), were tracked throughout the course. One month after initiating steroid therapy, her serum ALP (S-ALP) level decreased by 27.1% from baseline, while serum TRACP-5b (S-TRACP-5b) and serum ucOC increased by 34.4% and 52.3%, respectively, although BMD remained unchanged. Two months into treatment, she developed thoracic vertebral fractures (VFs) and was diagnosed with GIOP, prompting the initiation of oral alendronate sodium hydrate. Following the introduction of antiresorptive therapy and a reduction in GC dosage, both S-ALP and S-TRACP-5b levels returned to baseline by six months, accompanied by an 8.8% increase in BMD compared to the one-month level. No further fractures were observed after the cessation of antiresorptive treatment, which was guided by serial monitoring of BMD and BTMs. This case underscores the association of VFs with a decline in bone formation markers and elevations in bone resorption and matrix-related markers and demonstrates how BTMs reflecting bone quality can aid in determining the optimal duration of antiresorptive treatment in pediatric patients with GIOP.

## Introduction

Glucocorticoids (GCs) are essential medications for treating various renal diseases in children and are known to yield favorable clinical outcomes [1]. However, their use can lead to GC-induced osteoporosis (GIOP), which may result in vertebral fractures (VFs) [2]. These fractures can limit children’s ability to engage in physical activity - an important factor in both mental development and physical growth. Despite this, there is currently no established clinical management for GIOP in children, and more data on the condition in pediatric patients is still awaited [3-6].

A 2001 National Institutes of Health consensus conference defined osteoporosis as a skeletal disorder characterized by compromised “bone strength,” which is primarily determined by bone mineral density (BMD) and bone quality [7]. BMD is typically evaluated via X-ray, while bone quality is assessed clinically using serum bone turnover markers (BTMs), such as alkaline phosphatase (ALP), tartrate-resistant acid phosphatase 5b (TRACP-5b), and undercarboxylated osteocalcin (ucOC) [8].

In this report, we present the case of a girl who was treated with GCs for Henoch-Schönlein purpura nephritis (HSPN) and subsequently developed VF as a result of GIOP. Her BMD and BTMs were monitored over a two-year period during steroid therapy.

This observational study suggests that the combined assessment of three BTMs may serve as a predictive marker for VF. Moreover, these markers could help guide decisions regarding the appropriate duration of antiresorptive therapy.

## Case presentation

A six-year-old girl was diagnosed with IgA vasculitis and was treated with prednisolone (PSL). At that time, her body weight was 20 kg. The initial dose was 1 mg/kg/day for three weeks, after which it was gradually reduced. Upon tapering the PSL dose, proteinuria (urinary protein/urinary creatinine: 2-3 g/gCr) and hematuria (>100/HPF) were detected. A renal biopsy was performed to assess her renal condition. The pathological findings were consistent with grade IIIb of the International Study of Kidney Disease in Children classification for HSPN. Consequently, methylprednisolone pulse therapy (MPT, 500 mg/day) was administered for three consecutive days, followed by daily oral PSL (25 mg/day) and mizoribine (120 mg/day) for a total of three courses when she was seven years old.

By that time, she had been receiving oral steroid therapy for one year (with a total daily PSL dose of 0.6 mg/kg before MPT). Due to a recurrence of purpura and abdominal pain, the daily PSL dose was increased to 25 mg just before initiating MPT. After MPT, the GC dose was gradually tapered. The trends in BMD and serum BTMs - including serum ALP (S-ALP), serum TRACP-5b (S-TRACP-5b), and serum uCOC (S-ucOC) - were monitored over a two-year period during GC therapy.

**Figure 1:**
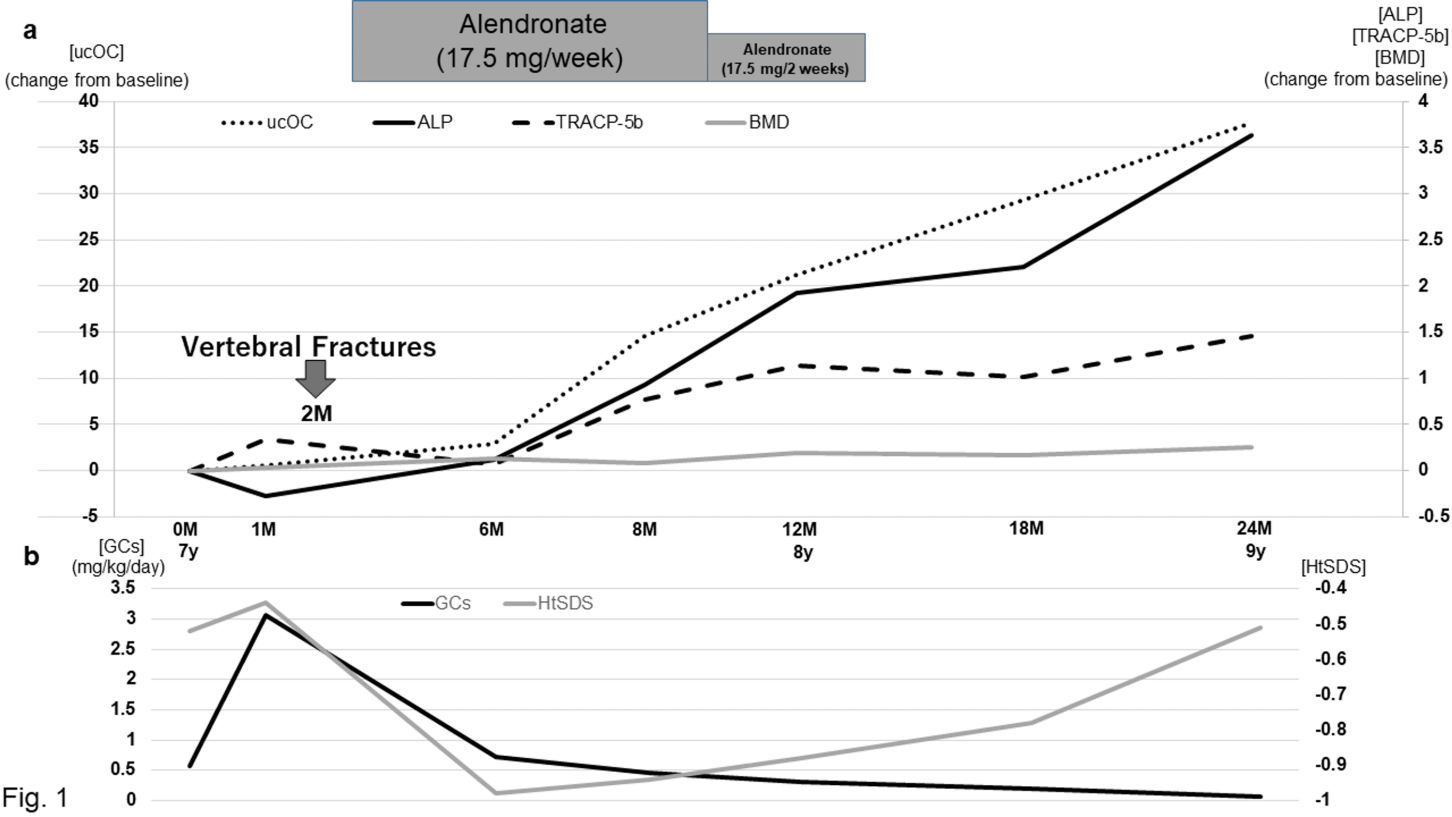
Trends in BMD and BTM in a girl with HSPN who developed VFs under GC treatment (a) Changes in BMD at the lumbar spine, S-ALP, S-TRACP-5b, and S-ucOC from baseline (0M) following MPT over two years. (b) Total daily GC dose (mg/kg), accumulated between each evaluation time point for bone strength and HtSDS following methylprednisolone pulse therapy over two years. BMD, bone mineral density; BTM, bone turnover marker; GC, glucocorticoid; HSPN, Henoch-Schönlein purpura nephritis; HtSDS, height standard deviation score; MPT, methylprednisolone pulse therapy; S-ALP, serum alkaline phosphatase; S-TRACP-5b, serum tartrate-resistant acid phosphatase 5b; S-ucOC, serum undercarboxylated osteocalcin; VF, vertebral fracture

Her clinical characteristics at baseline (0M, defined as just prior to MPT, i.e., one year after the initiation of GC treatment for IgA vasculitis) are shown in Table 1. Her body weight at baseline was 27.3 kg. Trends in BMD and BTMs during GC treatment are presented in Figure 1a. The total daily GC dose (mg/kg) accumulated between each bone strength evaluation time point, along with the height standard deviation score over the two-year period following MPT, is shown in Figure 1b.

**Table 1:** Clinical characteristics at baseline The PSL dose represents the cumulative dose over the one-year period prior to steroid pulse therapy. The baseline time point corresponds to just before the initiation of steroid pulse therapy, which was one year after starting PSL treatment for IgA vasculitis. BMD, bone mineral density; Ca, calcium; Cr, creatinine; P, phosphorus; PSL, prednisolone; S-ALP, serum alkaline phosphatase; S-TRACP-5b, serum tartrate-resistant acid phosphatase 5b; S-ucOC, serum undercarboxylated osteocalcin; Up/Ucr, urinary protein/urinary creatinine

Parameter	Value
S-ALP (U/L)	214
S-TRACP-5b (mU/dL)	532
S-ucOC (ng/mL)	0.86
BMD (g/cm²)	0.526
BMD Z-score	−2.1
Serum Cr (mg/dL)	0.33
Serum Ca (mg/dL)	9.2
Serum P (mg/dL)	4.2
Up/Ucr ratio	2.8
PSL dose (mg/kg/day)	0.6

One month after beginning MPT (1M), the level of S-ALP, a marker of bone formation, decreased by 27.1% from baseline (0M: prior to MPT). The levels of S-TRACP-5b, a marker of bone resorption, and S-ucOC, a bone matrix-related marker, increased by 34.4% and 52.3% from baseline, respectively. However, BMD did not change (Figure 1a). Two months after starting MPT (2M), the patient was taking oral PSL (20 mg/day) and developed VFs in her thoracic spine (T5 and T6) on MRI after jumping rope. As a result, oral alendronate sodium hydrate (17.5 mg/week) and alfacalcidol (0.5 μg/day) were started, and the daily PSL dose was reduced to 18 mg. At six months (6M), both S-ALP and S-TRACP-5b levels had recovered to baseline, and S-ucOC level increased by 295% from baseline. In line with the recovery of bone formation and resorption marker levels, BMD increased by 8.8% from that at 1M (Figure 1a). At eight months (8M), S-ALP, S-TRACP-5b, and S-ucOC levels had increased by 72.4%, 66.3%, and 294% from 6M, respectively, and BMD remained above baseline. MRI showed no further deformities. Consequently, the antiresorptive treatment was tapered every two weeks. At 12 months (12M), S-ALP, S-TRACP-5b, S-ucOC levels, and BMD had increased by 51.7%, 20.9%, 42.5%, and 9.8% from 8M, respectively. The patient’s growth velocity standard deviation score (SDS) was +1.64 during the six-month period from 6M to 12M (Figure 1b). This clinical course suggested that her bone turnover was becoming more active, and the antiresorptive treatment was discontinued. At 24 months (24M), S-ALP, S-TRACP-5b, S-ucOC levels, and BMD remained higher than baseline. Her growth velocity SDS was +2.92 during the most recent 12-month period (12M-24M) (Figure 1a, 1b).

Her growth acceleration during the observational period was not accompanied by the development of secondary sexual characteristics. No further VFs occurred after the termination of antiresorptive treatment, based on serial BMD and BTM findings.

## Discussion

This case highlights two important points regarding the management of GIOP in a child with HSPN. First, an increase in both S-TRACP-5b and S-ucOC levels, along with a decrease in S-ALP levels, may be associated with VF. Second, a consistent increase in S-ALP, S-TRACP-5b, and S-ucOC levels, with BMD remaining above baseline, could signal the possibility of discontinuing antiresorptive treatment in a child with GIOP. To the best of our knowledge, this is the first report to describe the trends in BMD and BTMs throughout the clinical course of HSPN under GC treatment over a two-year period.

In renal diseases, several studies have reported nephrotic children experiencing VFs due to GIOP [9]. However, there have been no serial studies examining the effects of GCs on bone strength using BMD and three different types of BTMs.

At 1M, the S-ALP level had decreased by 27.1% from baseline, which we hypothesize reflects suppression of bone formation caused by GCs. In contrast, the 34.4% increase in S-TRACP-5b levels at 1M may reflect increased bone resorption due to GCs [10]. The S-ucOC and S-ALP levels changed in opposite directions until 1M. The increase in S-ucOC indicates bone matrix deterioration [11] and is a known fracture risk factor [12], while the 23.6% decrease in S-ALP suggests suppression of bone formation [13]. These opposite trends until 1M indicate deterioration in bone quality [11,13]. While BMD did not decrease, this does not necessarily imply that bone strength was normal, as the changes in BTMs suggested a decline in bone quality. VF occurred at 2M.

Following the initiation of antiresorptive treatment and a reduction in the PSL dose, S-ALP and S-TRACP-5b levels returned to baseline by 6M. The total daily PSL dose was reduced from 1M to 6M, from 3.1 to 0.72 mg/kg, which likely stimulated bone formation, as evidenced by the increase in S-ALP, while antiresorptive treatment suppressed bone resorption, reflected in the decrease of S-TRACP-5b. Both S-ALP and S-ucOC levels changed in the same direction as the GC dose was reduced, which may indicate accelerated bone formation [6], as suppression of bone turnover led to enhanced bone turnover with the reduction of GC dose [6,14]. In line with these changes in BTMs, BMD began to increase. From 6M to 24M, as the total daily PSL dose decreased from 0.7 to 0.06 mg/kg, S-ALP, S-TRACP-5b, and S-ucOC levels increased, and BMD remained above baseline. These findings suggest that reducing the PSL dose may activate bone turnover. We observed accelerated bone turnover (i.e., a consistent increase in BTMs and BMD remaining above baseline), which indicated improved bone strength, and we subsequently discontinued antiresorptive treatment. The patient’s growth improved significantly during this period, and VF did not recur after the termination of antiresorptive treatment. Therefore, a consistent increase in BTMs and BMD remaining above baseline may allow for the discontinuation of antiresorptive treatment in children with GIOP. A limitation of this study is that BMD or serum BTMs at baseline (0M) were measured during GC treatment for IgA vasculitis (with a total daily PSL dose of 0.6 mg/kg). Another limitation is that BMD and BTMs were not measured at 2M, when VFs occurred.

## Conclusions

An increase in both S-TRACP-5b and S-ucOC levels, accompanied by a decrease in S-ALP levels, may be associated with VF. Additionally, a consistent increase in S-ALP, S-TRACP-5b, and S-ucOC levels, with BMD remaining above baseline, could facilitate the termination of antiresorptive treatment in a child with GIOP. These findings could enhance the clinical management of GIOP in children. Further studies involving children under GC treatment are needed to confirm the generalizability of these results.
